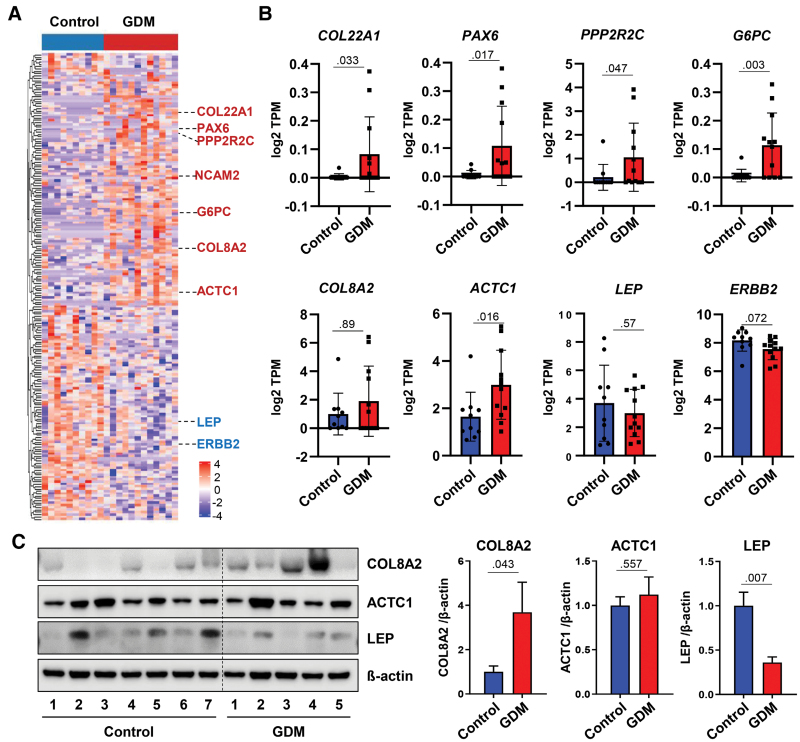# Placental transcriptome profiling and immune signature differences in pregnancies with and without gestational diabetes mellitus: An observational study: Erratum

**DOI:** 10.1097/MD.0000000000049792

**Published:** 2026-07-10

**Authors:** Yea Eun Kang, Seong Eun Lee, Joung Youl Lim, Ok Soon Kim, Jiyeon Yoon, Yewon Jung, Ju Hee Lee, Bon Jeong Ku, Mina Lee, Hyun Jin Kim

In the article “Placental transcriptome profiling and immune signature differences in pregnancies with and without gestational diabetes mellitus: An observational study,”^[1]^ which appears in Volume 105, Issue 21 of *Medicine*, figures 1 and 2 were inadvertently published in a cropped format. The complete versions of figures 1 and 2 have now been updated in the published article.

**Figure 1.**
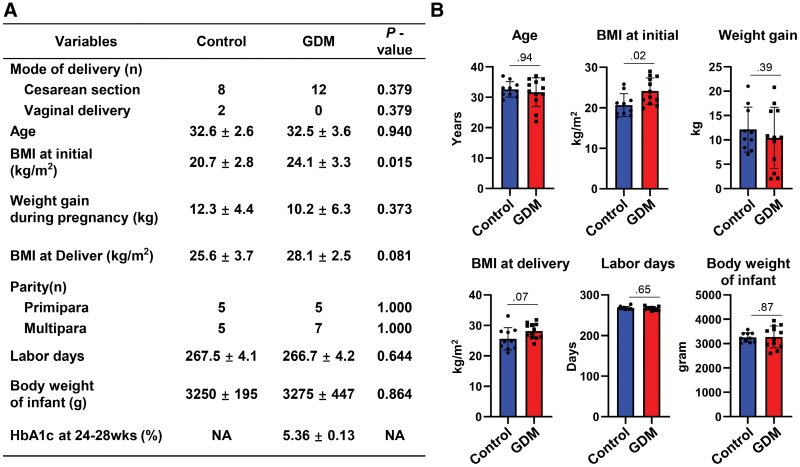


**Figure 2.**